# Environmental risk of Covid-19 recovery

**DOI:** 10.1177/0958305X221108493

**Published:** 2022-07-15

**Authors:** Mortaza Baky Haskuee, Ali Asgary

**Affiliations:** Disaster and Emergency Management, School of Administrative Studies, 177432 Faculty of Liberal Arts and Professional Studies, York University, Toronto, ON, Canada

**Keywords:** Covid-19, economic recovery, Kuznets curve, environmental risk

## Abstract

During Covid-19 pandemic world economy experienced negative growth rate, therefore energy consumption and consequently emission pollution decreased. According to Environmental Kuznets Curve, it is expected that energy consumption and emission pollution increase in response to Covid-19 economic recovery, even higher than its pre-pandemic level. The goal of this paper is to study the environmental risk of Covid-19 economic recovery. We use an Environmentally-Augmented Global Vector Autoregressive Model (E-GVAR) to trace dynamic effects of Covid-19 economic recovery on pollution emission. Using generalized impulse response functions (GIRFs), we investigated the effect of positive economic shocks in real per capita income in China and USA economies on total $CO_{2}$ equivalent emission pollution. The results show that positive economic recovery affects emission pollution significantly. China and emerging economies may experience high risk while Europe region is moderately affected by this positive shock. *A positive Economic Shock in China decrease pollution emission in USA* over time. It can be attributed to substitution effect of Chinese product in global market. Generally, our results demonstrate spillover effect of transition shocks from large economies to the rest of world and highlights the importance of linkages in the world economy.

## Introduction

Covid-19 pandemic has affected world economy negatively and caused slowdown in economic activities. The transportation sector is most affected by the COVID-19 due to the large-scale restrictions on mobility and aviation.^1,2^ Decrease in transportation demand and energy consumption resulted in emission pollution production. Iqbal et al.^3^ investigated how the COVID-19 pandemic reduces $CO_{2}$ emission and energy consumption. Structural changes, like the COVID-19 pandemic can affect energy markets, energy consumption and $CO_{2}$ emission.

Emission pollution trend has been increasing during the last two decades except for 2009 in response to 2007–2008 financial crisis and recession. Since the start of the pandemic, because of the decrease in energy consumption, emission production was reversed. Total $CO_{2}$ emissions from fuel combustion (Mt $CO_{2}$) decreased in 2020 by 4.8% at global level. Oil, coal, and gas contribute to emission pollution by 31%, 44% and 24% respectively.

Meadows et al.^4^ argue that economic growth has negative impact on environment while Dasgupta and Heal^5^ provides evidence on complementary relationship between economic growth and environment. Grossman and Krueger^6^ show that there is a non-linear relationship between economic growth and environmental degeneration can be presented in a bell-shaped curve, known as Environmental Kuznets Curve (EKC). It is asserted that at the beginning of economic growth, environmental degeneration rises and declines after it reaches its maximum level. Therefore, we expect that at the beginning of the Covid-19 economic recovery, the world economy will experience a sharp increase in environmental pollution emission. Because of the high degree of integration between world economies^1^, any change in leading economies-with high share in the world GDP would be immediately transmitted to the rest of the world. Investigations show that during the last decades growth in large economies such as China has had significant effect on world energy market and growth in China pushed not only the domestic energy consumption but also pushed up energy consumption in resource-based economies. Therefore, it is expected that economic recovery in a large country would be spilled over into other economies.

Since the Covid-19 began, many studies have been conducted to investigate macroeconomic effects of the crisis. McKibbin and Fernando^7^ applied a DSGE/CGE model to explore the global macroeconomic effects of the pandemic under different scenarios. Their results highlighted the importance of spillover effect. Bonadio et al.,^8^ using data for 64 economies investigated the effect of Covid-19 crisis on the world supply chains. Baqaee and Farhi^9^ used a multi-sector model with input-output linkages, nominal wage rigidities and bounded policy rate to study non-linearities in response to Covid-19 pandemic. The model accommodated to the USA data and the results show how negative effects of crisis could be magnified through nonlinearities. Milani^10^ applied a GVAR model and demonstrated how important are linkages to amplify the negative effects of crisis on unemployment. The aim of this paper is to investigate the effect of Covid-19 economic recovery on environmental pollution assuming that with vaccination and other measures countries gradually shift to the recovery process. We apply an Environmentally-Augmented Global Vector Autoregressive Model (E-GVARX) to study the dynamic effects of positive economic shocks in large economies, USA, and China, on total $CO_{2}$ equivalent emission pollution at global level. The paper is structured as follow. Section two reviews the literature focusing on the relation between economic growth and environment. Section three is dedicated to data and methodology. Section four presents the model estimations results. Section five concludes the paper.

## Literature review

Energy is considered as a factor of production and is essential for the world economy to function, thus economic growth is highly correlated to energy consumption.^11,12^ The relation between economic growth and energy consumption is well documented.^13–16^ U-shaped relation between economic growth and environmental degradation, well-known as EKC first examined by Grossman and Krueger.^6^ The concept, explains that at the initial stage of the economic growth, environment will be degraded and improvement will happen over time. According to the Environmental Kuznets Curve, there is a non-linear relationship between income level and environmental pollution emission. In the early stages of economic growth, emission pollution goes up and because of technological progress in energy appliances, the level of pollution produced at each level of income decreases with increase in per capita income. Therefore the pollution per capita decreases over time after passing its maximum level. The implication of this theorem is that, after any crisis, during the recovery process and with a positive shock in per capita income, the level of pollution will jump to a level higher than its pre-crisis level. Meanwhile, any positive shock in leading economies like China and the the USA could result in increase in energy consumption and $CO_{2}$ production.

Since the beginning of the pandemic, a series of studies have examined different aspects of this crisis. Studies show that this pandemic has affected economies in different ways including major decline in value chain, production, sales and employment rates.^17^ A few studies emphasize on the positive effects of Covid-19 on environment.^18–20^ These studies highlighted that Covid-19 has decreased the level of $PM_{2.5}$, $PM_{10}$, $NO_{2}$, and CO levels but not $SO_{2}$ and $O_{3}$ levels.

Investigations also provide evidence on reduction of $NO_{2}$ at global level.^21–23^ It is estimated that about 50 percent drop in CO and $NO_{2}$ emission in China happened due to shut down in heavy industries. In European metropolises, $NO_{2}$ emission dropped from 30–60%.^24^ In the USA, the $NO_{2}$ emission reduced by 25.5% during the pandemic.^25^ The level of $NO_{2}$ reduced from 4.5 pbb to 1 pbb across the province of Ontario, Canada.^26^ Sao Paulo of Brazil experienced a 54.3 percent decrease of $NO_{2}$.^27^ World Bank^1^ report predicts positive economic growth for almost all economies in 2022. Crucial recovery efforts and stimulus packages to support them focus on economic recovery, growth, and targeting a more resilient economy. Appearance of different vaccine platforms also brought trust into businesses and made positive forecasts. Any positive change in level of economic activities would increase energy consumption and finally emission pollution. It can be expected that any improvement in the status of the world economy, could result in increase in energy consumption and consequently pollution emission. The risk of increase in Green House Gases (GHGs) after the economic recovery cannot be ignored. Emission may increase in response to economic recovery, and the level of pollution may be much higher than its pre-pandemic level. Therefore, to design optimal policies to control environmental pollution and degradation, it is necessary to measure the amount of increase in GHGs in response to economic growth during the Covid-19 recovery.

## Data and methodology

To achieve the objective of this study we used an environmentally augmented version of the GVAR model of Dées, di Mauro, Pesaran, and Smith^8^ named DdPS.^28^ GVAR is a global modeling framework for analyzing the international macroeconomic transition of shocks considering links between different economies, originally proposed by Pesaran et al.^29^ and developed by Dees et al.^28^ as a tool for credit risk analysis and applied in numerous other studies. It is particularly suitable for analyzing the transmission of shocks from one market, country, or region to other markets and economies. GVAR has a number of interesting attributes that makes it an ideal method for our analysis; (1) the GVAR is able to capture complex national an international interactions and inter-dependencies; (2) it has theoretical consistency for long-run relationships and data consistency in short-run; (3) it handles dimensionality by assuming that most foreign variables are weakly exogenous; (4) it allows for country models to be estimated separately and aggregated later; (5) it can be used for large or small number of countries or different groups of countries” (p.13).^30^

GVAR applications in researches include bank stress testing; analysis of China's growing importance for the rest of world economy;^31^ international macroeconomic transmission of weather shocks; consequent impacts of oil price shocks^32^ as a result of oil supply^33,34^ and demand driven shocks^35^ as well as forecasting.^36,37^ Chudik et al.^38^ developed a threshold augmented dynamic multi country model to analyze macroeconomic impacts of Covid-19. Chudik et al.^39^ applied a threshold-augmented Global VAR model to quantify the macroeconomic effects of countries’ discretionary fiscal actions in response to the Covid-19 pandemic and its fallout.

### Global energy consumption and emission production

**Figure 1.**
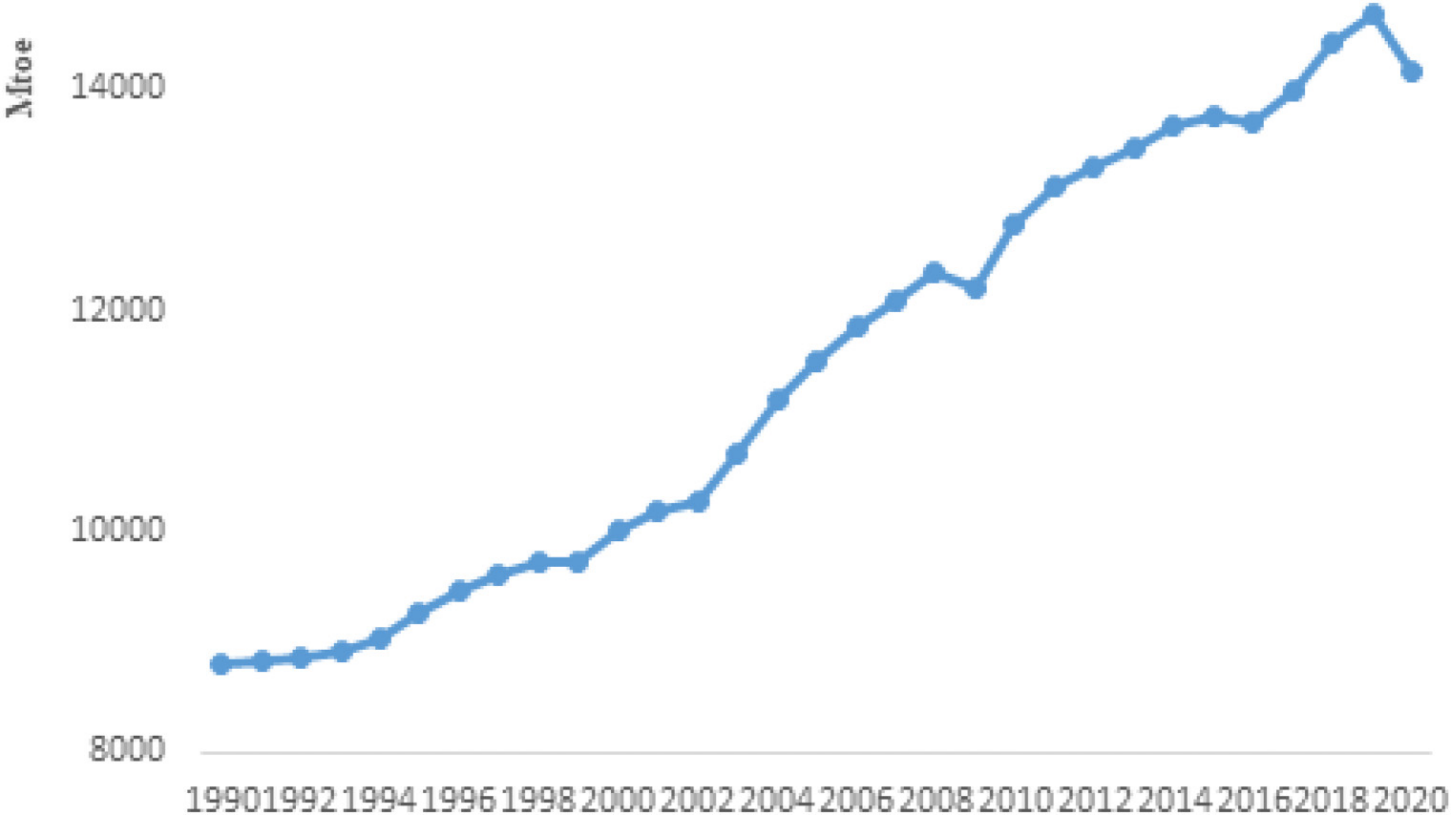
World energy consumption 1990–2020.

Source: Global Energy Statistical Yearbook 2021.

Figure 1 illustrates world energy consumption over the period of 1990–2020.Global energy consumption growth was 2% on average over the period of 2000–2018. In 2019, and alongside to Covid-19 outbreak, it fell to 0.8%. In 2020, global energy consumption decreased by 4%, due to lockdown measures and transport restrictions. Fall in energy consumption growth was not homogeneous between economies. Although it fell in most countries, China, the largest energy consumer which consumed 24% of the global energy in 2020, and rapidly recovered from the Covid-19 crisis had a 2.2% growth in energy consumption, but the momentum was lower than annual average over the 2008–2018 period and +3.4% in 2019.^40^

In the USA, growth of energy consumption was 0.5% on average over the period of 2000–2018, which dropped to −7.4% in 2020, due to Covid-19 pandemic. North America's response to the pandemic in terms of energy consumption was the same as the USA. Advanced economies including EU members, Japan, and Canada experienced a 7% decrease in energy consumption. Emerging economies including India, South Korea, and in less developed economies namely Saudi Arabia experienced 1.3% decrease in total energy consumption. The growth rate of energy consumption has been around −2% in Australia and Brazil during the pandemic period. In the Middle East and North Africa (MENA), energy consumption also contracted by 1.2%, while it has increased by 4.2% per annum over the 2000–2018 period. The Latin America's rate of energy consumption over 2019–2020 was −6.9% much lower than its annual average growth during the 2000–2018 period.^40^

**Figure 2.**
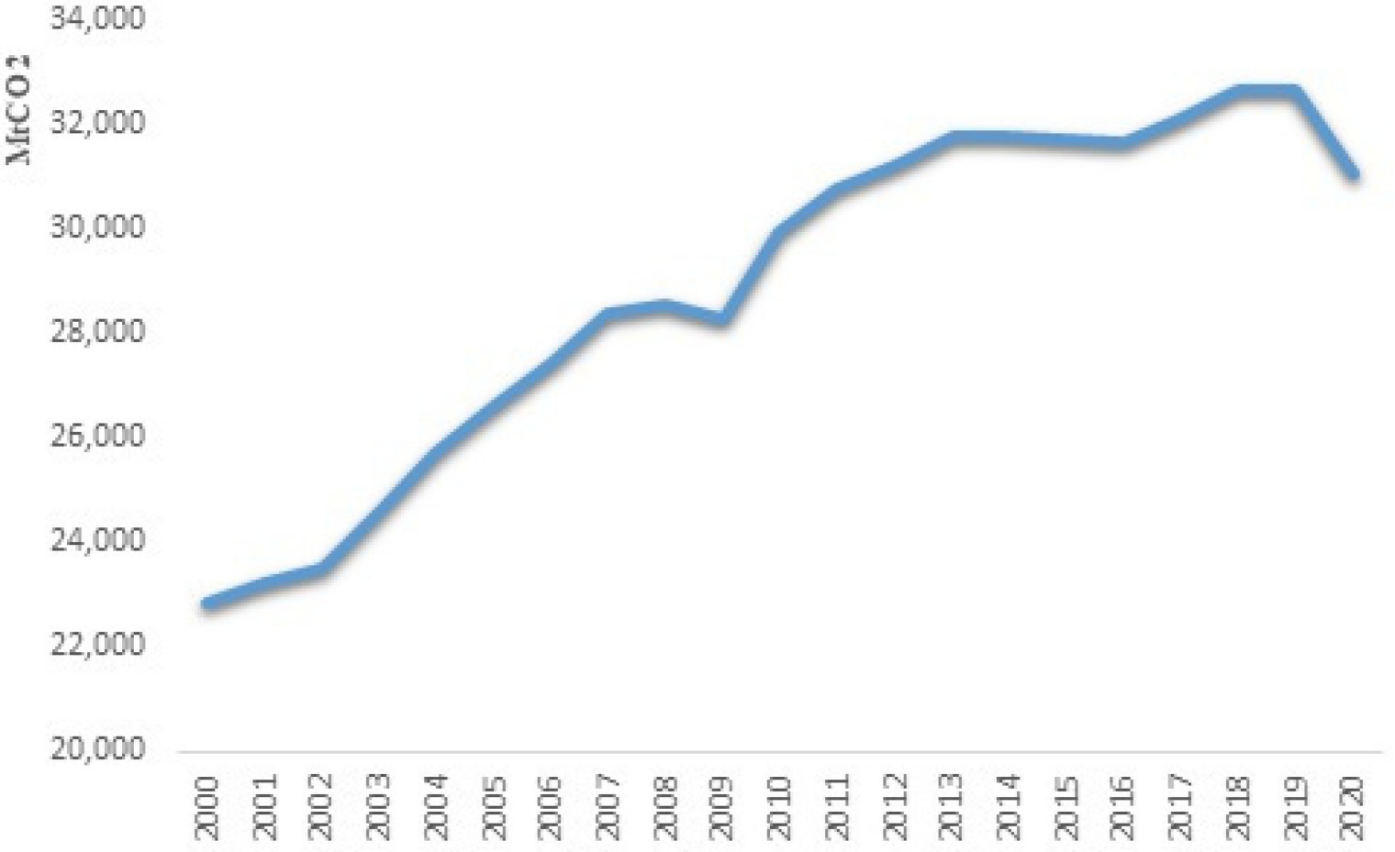
Total $CO_{2}$ emissions from fuel combustion 1990–2020.

Source: Global Energy Statistical Yearbook 2021.

Figure 2 shows total $CO_{2}$ emissions over the period of 2020–2020. As it can be seen, over this period total $CO_{2}$ emissions were increasing except for 2008–2009 financial crisis. In 2020, $CO_{2}$ emissions fell by 4.9% in response to decrease in economic activities; however, the momentum was below its 2012 level.^40^ Widespread lockdown measures, transport restrictions and the economic slowdown significantly reduced oil consumption in the transport sector. $CO_{2}$ emissions also contracted in the power sector, because of the lower electricity demand and the continued decline of the carbon factor ($CO_{2}$ emissions per kWh produced), mainly due to fuel switching from coal to gas and the rising share of renewable energy in the global power mix.

$CO_{2}$ emission reduced by 11% in the USA and Europe. Significant cuts in $CO_{2}$ emission occurred in Germany, Spain, and the UK, due to a much lower coal-fired power generation and higher $CO_{2}$ prices in 2020. India produced 5.5% less $CO_{2}$, due to the lower coal-fired power generation and oil product consumption. In Russia and Canada, the main source of $CO_{2}$ reduction was reduced power generation and sharp drop in oil production and consumption. In Japan and South Korea, $CO_{2}$ emission reduced by 6.6% and 6.2% respectively, mainly because of increased share of renewable in the power mix. $CO_{2}$ emission production was reduced in Latin America (mainly in Mexico, Brazil and Argentina), Africa, (strongly in South Africa) and in the Middle East (notably in Saudi Arabia, where oil consumption decreased significantly). On the contrary, $CO_{2}$ emissions rose for the fourth year in a row in China (+1.6%), due to a rapidly recovering energy demand and a steady coal-fired power generation, despite a new surge in renewable power generation. China accounted for 31% of global $CO_{2}$ emissions in 2020.

### The country-specific VARX* models

The GVAR model applied in this research is an environmentally-augmented version of the DdPS^28^ model, which integrates environmental energy related variables into the basic model. The model accepts four domestic variables of DdPS^28^ and three global variables. Therefore, our model variables include total per capita $CO_{2}$ equivalent pollution emission, $CO_{2}it$, energy intensity index, intsit, log real GDP, yit, rate of inflation, dpit, short-term interest rate, rit, long-term interest rate, lrit (domestic variables and their coexistence foreign variables), oil prices, $poil_{t}$, agricultural raw material, $pmat_{t}$ and metal prices, $pmetal_{t}$ (global variable).

To adapt the GVAR model for the analysis of the environmental risk of the Covid-19 economic recovery, and transition of economic growth in large economies; China and the USA on $CO_{2}$ pollution, we assume that there are N + 1 countries in the global economy, indexed by i=0,1,…,N, where 0 is a reference country, namely a large economy. The models consist of a number of country-specific macroeconomic variables collected in the vector $X_{it}$ and their counterparts foreign variable which are weighted average of macroeconomic variables that the wights are share of counter party in total trade of each country, as well as the above mentioned global variables over time, t = 1,2,…,T and across the “N + 1” countries. Each country includes a set of domestic, foreign specific, and global variables which do not vary across economies. This is a large-scale complex system of equations that needs a large data base to estimate the parameters of the model. Therefore, country-specific models are estimated separately treating foreign and global variables as weakly exogenous. This method of solving for ‘curse of dimensionality’, goes back to Fleming,^41^ Mundell^42^ and Dornbusch.^43^ A country-specific VARX*(p, q) structure given by:

(1) $$X_{it}=a_{i0}+a_{i1}t+\overset{p}{\underset{\;j=1}{\sum }}\;\phi_{i,j}X_{i,t-j}+\overset{q}{\underset{l=0}{\sum }}\;\Lambda_{i,l}X_{i,t-l}^{*}+\epsilon_{it}$$

where $X_{it}$ stands for $k_{i}\times 1$ vector of domestic variables (which includes total per capita $CO_{2}$ equivalent pollution emission, $CO_{2}it$, energy intensity index, intsit, log real GDP, yit, rate of inflation, dpit, short-term interest rate, rit, long-term interest rate, lrit and their coexistence foreign variables in our model), $X_{it}^{*}$ is $k_{i}^{*}\times 1$ vector of foreign and global variable (oil prices, $poil_{t}$, agricultural raw material, $pmat_{t}$ and metal prices, $pmetal_{t}$ in our model). $u_{it}$ is a serially uncorrelated and cross-sectionally weakly dependent process with mean 0 and a nonsingular covariance matrix, $\underset{ii}{\sum }=(\sigma_{ii,ls})$ which shows contemporaneous dependance of shocks in country *i* on shocks in country *j*. This assumption is necessary for specification of related foreign variables to domestic variables. Foreign- specific variables are weighted average of corresponding domestic variables of all countries, with country-specific weights equal to $X_{it}^{*}=\overset{N}{\underset{\;j=1}{\sum }}\;W_{it}X_{it}$. Glick and Rose^44^ discuss the importance of trade links in the analysis of transmission of crisis and shocks between economies. In a more general framework, it is better to allow for change in weights over time to capture specific movement in the geographical patterns of trade and capital outflows. In this paper we assume fixed weights which are 10 years average of trade flows over time. To construct a GVAR model from the country-specific models, a $\left(k_{i}+k_{i}^{*}\right)$ vector is defined as follow:

(2) $$Z_{it}=\left(\begin{array}{c} X_{it}\\ X_{it}^{*} \end{array}\right)$$

then, equation is rewritten as

(3) $$A_{i0}Z_{it}=\overset{p}{\underset{l=1}{\sum }}\;A_{il}Z_{il,t-l}+\epsilon_{it}$$

where $A_{i0}=(I_{ki}-\Lambda_{i0})$, $A_{il}=(Q_{il},\Lambda_{il})$ for l=1,2,…,p and $p=\mathrm{ma}x_{i}(\;p_{i},q_{i})$ and $\Phi_{il}=0$ for $l>p_{i}$. Econometric theory to estimate $VARX^{*}(\;p,q)$ developed in Harbo et al.^45^ and Pesaran et al.^46^ Error correction representation form of (3) can be written as;

(4) $$\Delta X_{it}=\Lambda_{i0}\Delta X_{it}-\Pi_{i}Z_{i,t-1}+\overset{p}{\underset{l=1}{\sum }}\;H_{il}Z_{il,t-l}+\epsilon_{it}$$

whrere, Δ=1−L is difference operator, $\Pi_{i}=A_{i0}-\overset{p}{\underset{l=1}{\sum }}\;A_{il}$ and $H_{il}=-(A_{i,l+1}+A_{i,l+2},\ldots ,A_{i,l+p})$ Equation (4) represents cointegration relation between domestic variables as well as between domestic and foreign variables in each country-specific model. Since most macroeconomic variables are integrated of degree 1, I(1), then rank of $\Pi_{i}$ matrix indicates the number of cointegrating vectors, which can be decomposed into $\Pi_{i}=\alpha_{i}.\beta_{i}$, that $\alpha_{i}$ is $k_{i}\times r_{i}$ full column rank loading matrix and $\beta_{i}$ is $(k_{i}+k^{*})\times r_{i}$ full column rank matrix of cointegration vector.^47^ To trace the effect of positive economic shocks on environment, we use impulse response functions.

**Figure 3.**
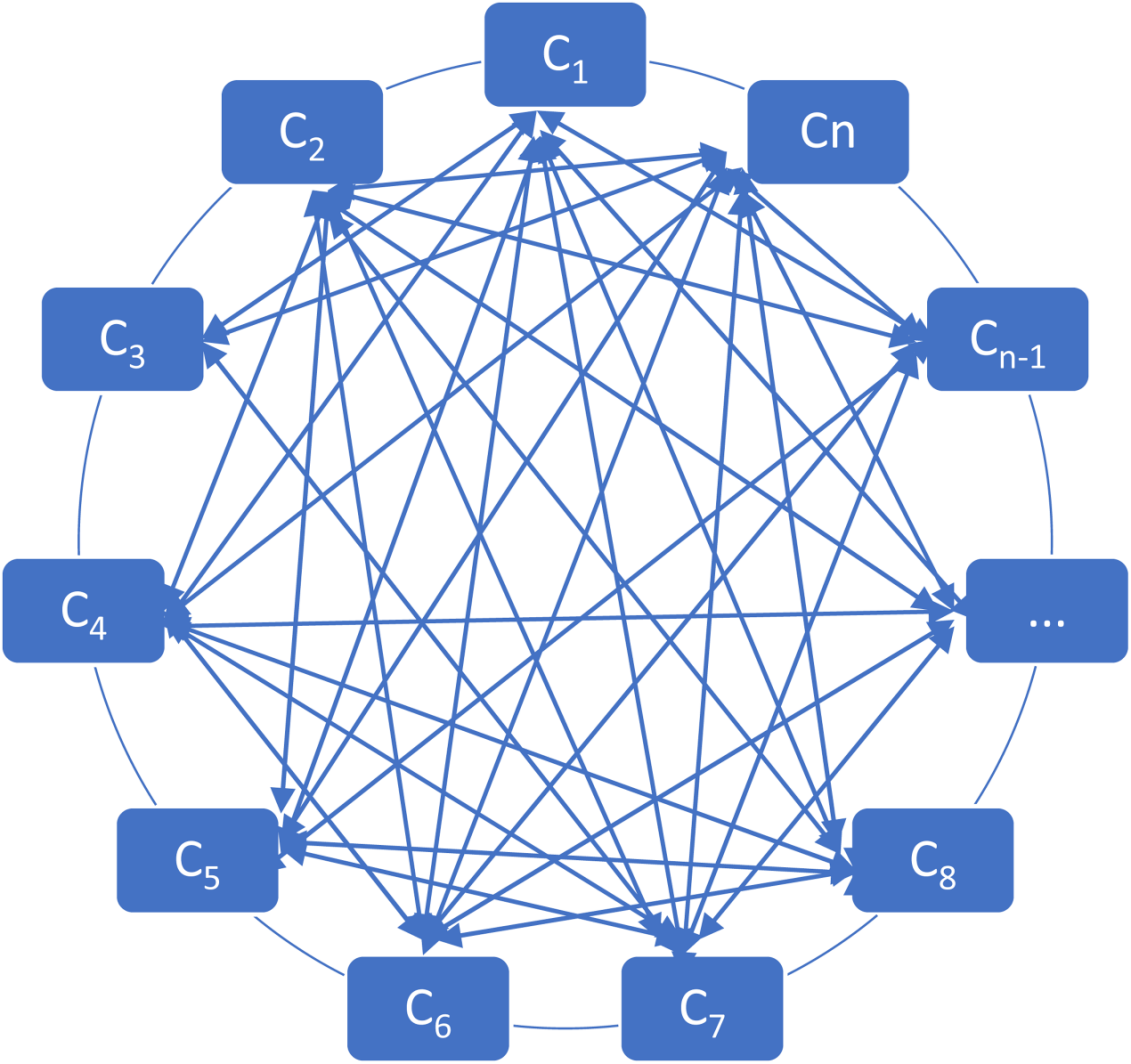
Schematic framework of n + 1 countries GVAR model.

Figure 3 shows schematic framework of N + 1 countries GVAR model which illustrates interactions between countries via a set of variables. Each array shows two-sided effect between each country.

### Data

The research uses latest updated version of the Global VAR (GVAR) Quarterly Dataset, for the Ddps,^28,48^ including quarterly macroeconomic variables for 33 economies over 1979Q2 to 2019Q4. Variables are included in most of the GVAR applications in the literature. These 33 countries cover more than 90 percent of the world GDP. Table 1 shows the countries and regions in the model. In this model large economies including United States, United Kingdom, China, and Japan considered separately. Other countries are categorized into different regions including Europe, Rest of Western Europe, Other Developed Countries, Rest of Asia, Latin America, and Rest of World. Switzerland, Sweden and Norway are not the members of European Union, therefore we put them into a separated European region. India, turkey, South Africa and Saudi Arabia categorized into Rest of World.

**Table 1.** Countries and regions in the model.

Europe	Rest of Western Europe	Other developed countries
Austria	Switzerland	Canada
Finland	Sweden	Australia
France	Norway	New Zealand
Germany	Latin America	Rest of Asia
Italy	Argentina	Indonesia
Netherlands	Brazil	Korea
Spain	Chile	Malaysia
Rest of World	Mexico	Philippine
India	Peru	Singapore
Turkey		Thailand
South Africa	China	Japan
Saudi Arabia	United States	United Kingdom

Database is constructed using data from Haver Analytics, International Monetary Fund's International Financial Statistics (IFS) database and Bloomberg. To make foreign variables of the model, we used weight matrix in updated version of the Global VAR (GVAR) Quarterly Dataset over 2014–2016. Weights are calculated based on bilateral trade between countries. We also extracted total per-capita $CO_{2}$ equivalent emission from fuel combustion and energy intensity index from $CO_{2}$ emission from Fuel Consumption, IEA, 2020 for 33 countries over 1979–2019. The research applies local quadratic interpolation method with average to change the annual data frequency into quarterly data. Thus X-12 ARMA seasonal adjustment method was applied to seasonally adjust the data. This method uses the X-11 seasonal adjustment method of Shiskin, Young and Musgrave^49^ and Dagum.^50^

### Model estimation and scenario analysis

Estimation of a system is not feasible unless for a moderate value of N. Unconstrained estimation of (33) country-specific model includes estimation of a large number of parameters which should not be greater than the number of observations. To solve this problem, and estimate the model feasibly, we assumed model-built fixed weights, $W_{ij}$, to make foreign variables. The weights are calculated using bilateral trade between countries over the period of 1980–2016. We assumed fixed weights which are the average weights over the study period. We also estimated country-specific parameters using a country-by-country approach rather than a simultaneous one. This approach also allows us to test those foreign and global variables that are weakly exogenous jointly.

Augmented Dickey- Fuller and Weighted Symmetric Dickey-Fuller unit roots tests introduced by Park and Fuller^51^ applies to test for trend and variance stationarity. Evidence provided by Pantula et al.,^52^ Leybourne et al.^53^ and Leybourne et al.^54^ show superior performance of the weighted symmetric test statistic compared to the standard ADF test or the GLS-ADF test proposed by Elliot et al.^55^ Optimum lags in tests are chosen by AIC and SBC. Test conducted for level, first order and second order differences of model's variables. The results show that model's variables have unit root at level for most of countries, but first difference of all variables are stationary. Therefore, most of variables are I (1). To decide on optimum lags of domestic, foreign and global variables, we used SBC/AIC criteria. Optimum lag for domestic variables differs between country-specific models. Optimum lags for domestic variable of Argentine, China, Indonesia, Korea, Peru, Philippine, South Africa, Saudi Arabia, Sweden, Thailand, Turkey and the USA is 2, while optimum lag of foreign and global variables in all country-specific is 1. We also conducted weak exogeneity test for foreign and global variables at the 5% significance level. Critical values of AIC, SBC and log likelihood are used for selecting the Order of the Weak Exogeneity regressions. The results show that weak exogeneity of all foreign and global variables can not be rejected at 95% confidence interval.

## Scenario analysis

Here we run some scenarios regarding the change in the world economy during the recovery from Covid-19 crisis. Any improvement in the world economy could result in energy consumption and pollution production. USA and China stand for more than half of the world GDP and contribute more than 50% of the world GHGs. Backward and forward linkages between these two large economies and spillover of growth in them have significant implication for the world economy, thus in this section we analyzed the effect of one standard error positive shock in per capita real GDP on total $CO_{2}$ equivalent emission.

### A positive economic shock in China

**Figure 4.**
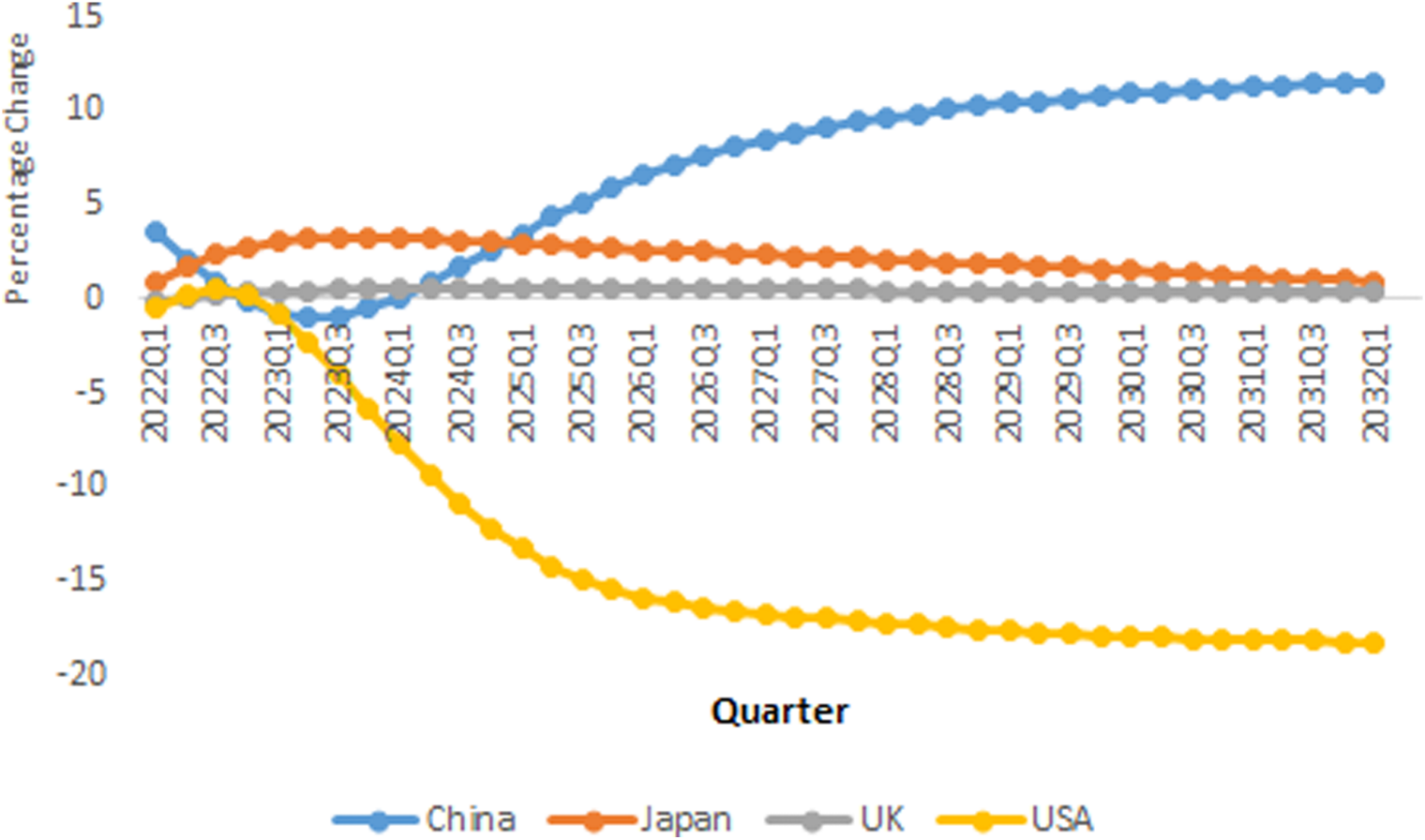
IRFs of $CO_{2}$ emission in response to one standard error shock in real GDP in China.

The research assumes one standard error positive shock in China's per capita GDP. The results show that the economic growth in China affects $CO_{2}$ pollution positively around the globe. This shock has a significant effect on pollution emission in China such that response of total $CO_{2}$ emission in China is three times more than the world level. Figure 4 compares response of total $CO_{2}$ pollution production in China, USA, Japan and UK in response to one standard error positive shock in per capita income. It shows Generalized Impulse Response Functions (GIRFs) for the time horizon 2021Q1–2032Q1. As it is shown, in the first three quarters, the shock increases $CO_{2}$ pollution. Although it decreases for the subsequent quarters, as it is shown, $CO_{2}$ emission will increase again and shows that China is exposed to recovery risk.

The USA economy's responses to positive shock in China is moderate at the beginning of the recovery period and after a while, the total $CO_{2}$ pollution emission will decrease. It can be attributed to the fact that the USA and Chinese economies are the main players in the world economy, and growth in China's economy will result in smaller share of the USA economy out of the world GDP. On the other hand, most of international and multinational companies are established in China. Growth in Chinese economy means production of more manufactured products in China, and more export to the USA. Therefore, demand in the USA economy is fulfilled by the Chinese products, which implies less pollution production in the USA. Meanwhile, the effect of a positive economic shock in China on $CO_{2}$ emission in Japan can be significant. Our results show that positive economic shock in China increases pollution emission in Japan. It shows that Japan is exposed to high environmental risk of the economic recovery. It could be attributed to relatively low economic growth rate in Japan during the past few years. Any economic recovery in the world economy resulted from a positive shock in China's economy will affect economic growth in Japan.

**Figure 5.**
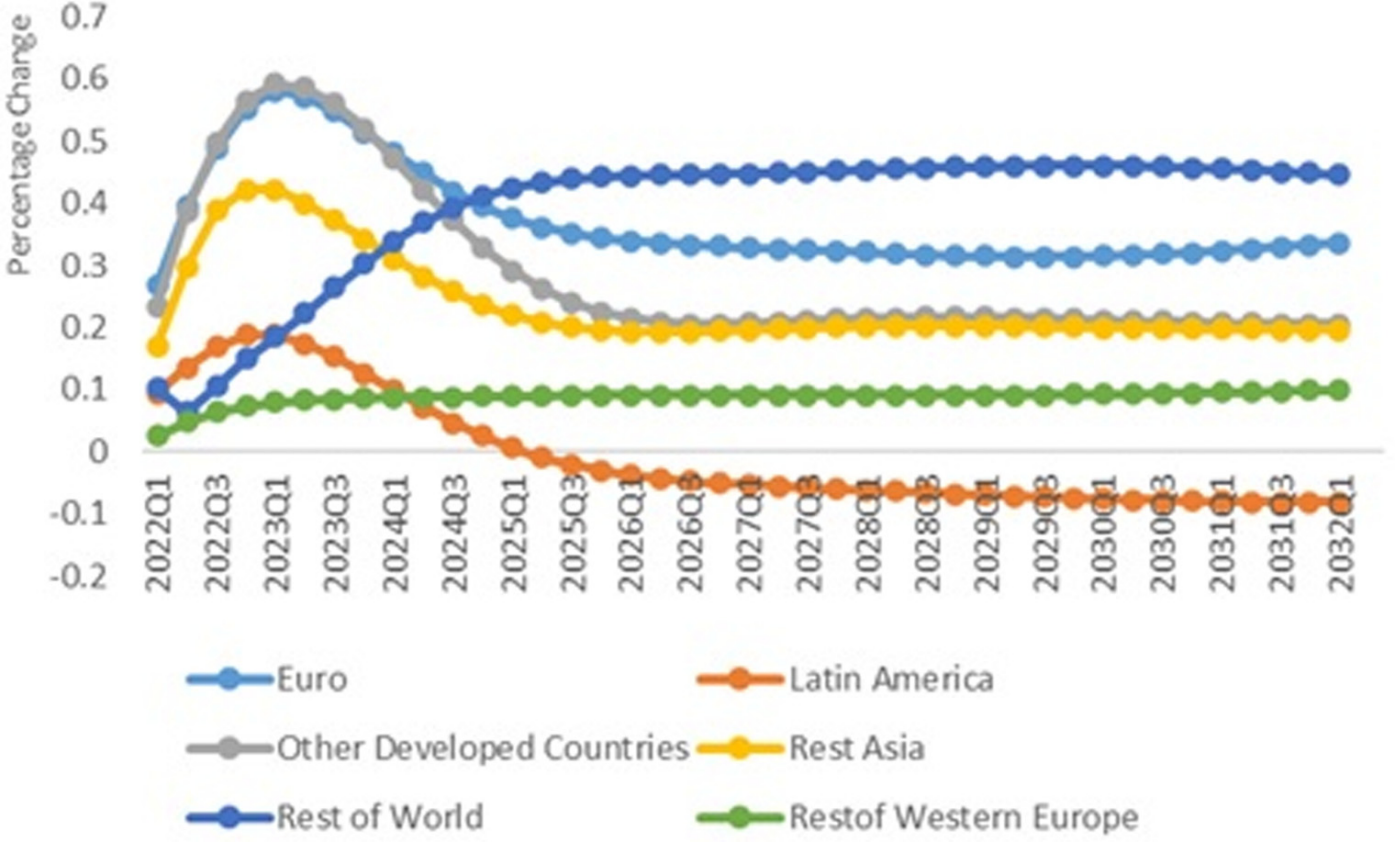
IRFs of $CO_{2}$ emission in response to one standard error shock in real GDP in China.

Our results show that the effect of this shock on the $CO_{2}$ emission pollution in the UK will be moderate. We also examined the effect of this shock at regional level. Figure 5 compares the effect of a positive economic shock in China on the emission in different regions; Europe (Austria, Belgium, Finland, France, Germany, Italy, Netherlands and Spain), Rest of Western Europe Countries (Switzerland, Sweden and Norway), Other Developed Countries (Canada, Australia and New Zealand), Rest of Asia (Indonesia, South Korea, Malaysia, Philippine, Singapore and Thailand), Latin America (Argentina, Brazil, Chile, Mexico and Peru) and Rest of the World (India, Turkey, South Africa, and Saudi Arabia). It illustrates GIRFs to that shock for the horizon of 2021Q1–2031Q1. The results show that this shock has a positive effect on total $CO_{2}$ emission in all regions.

Euro: Austria, Belgium, Finland, France, Germany, Italy, Netherlands, Spain; Rest of Western Europe: Switzerland, Sweden and Norway; Other Developed Countries: Canada, Australia and New Zealand; Latin America: Argentina, Brazil, Chile, Mexico and Peru; Rest of Asia: Indonesia, Korea, Malaysia, Philippine, Singapore and Thailand; Rest of World: India, Turkey, South Africa and Saudi Arabia.

In Europe, a positive shock in China's real GDP will affect $CO_{2}$ emission positively. The results show that the effect of this shock on emission pollution in Europe will be relatively larger with respect to other regions. Rest of Western Europe economies will be affected moderately by this shock. To figure out the effect of a positive shock on the real GDP of China on advanced economies in Rest of Western Europe region, the research also produced impulse response functions for Switzerland, Sweden and Norway. The results show that such shock has positive but relatively moderate effect on $CO_{2}$ emission in Switzerland and Sweden but has negative effect on Norwegian economy. Our results show that Other Developed Countries (Canada, Australia and New Zealand) stand in the second order after Europe. A positive economic shock in China's economy will increase $CO_{2}$ pollution emission in Latin American economies, but will decrease over time. In Argentina, Brazil, Mexico and Chile, the shock will increase pollution emission significantly, while its effect on $CO_{2}$ pollution emission in Peru will be moderate. The reason behind this could be because of the structure of these emerging economies. Growth in China will increase demand for goods in Latin American economies which will result in more pollution. This shock will affect emission pollution in Rest of Asia positively and the level of increase in pollution at the beginning of recovery will be higher than the Latin American countries. It can be attributed to the linkages between industries in China and the rest of Asia. The behavior of Rest of the World to this shock is different for mother regions. At the beginning of the recovery, pollution emission will increase moderately in this group of countries, but it will be increasing over time such that in long run the rate of response to shock will be higher than other regions.

### A positive economic shock in the USA

**Figure 6.**
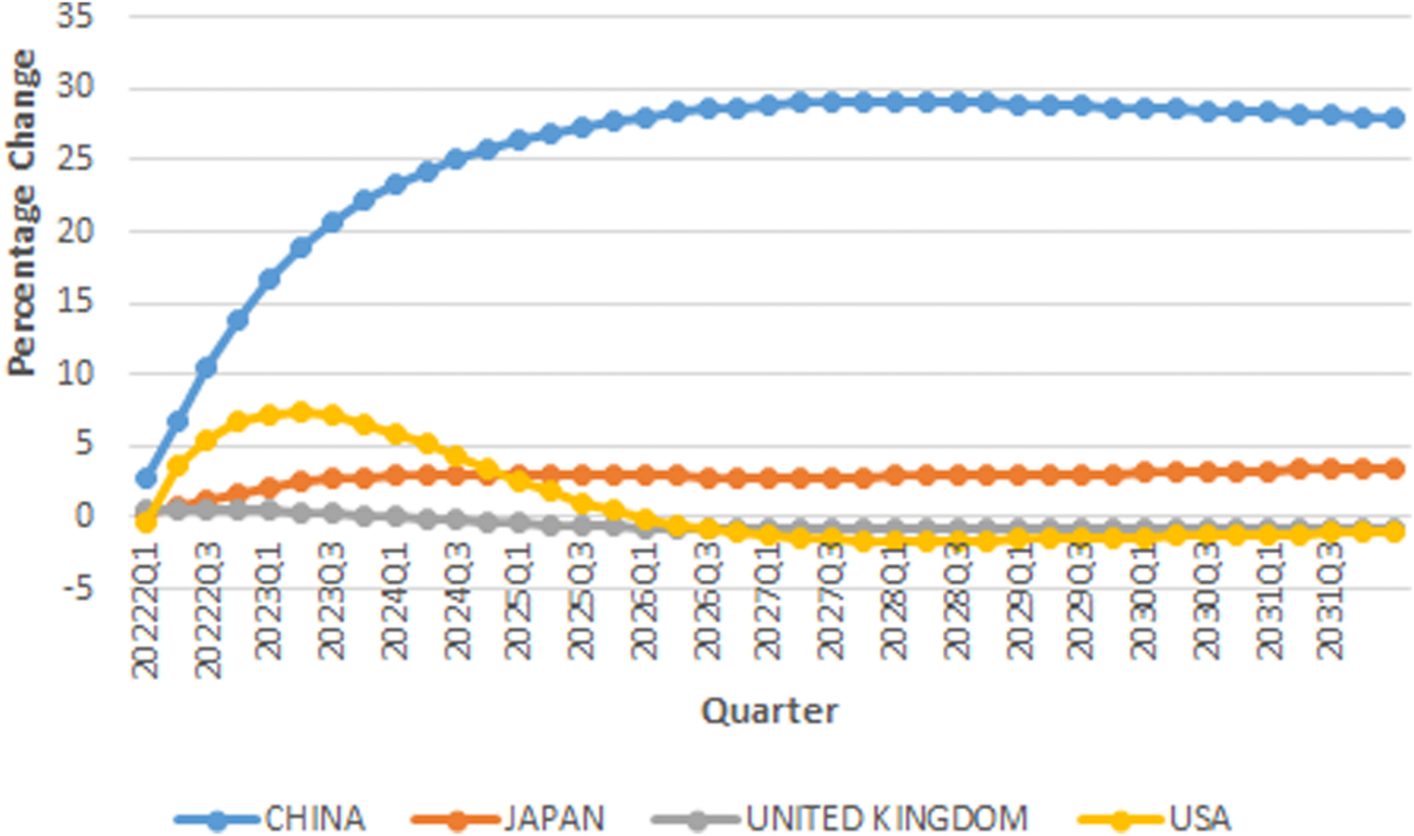
IRFs of $CO_{2}$ emission in response to one standard error shock in real GDP in USA.

The model assumes one standard error positive shock in the USA per capita real GDP to investigate the environmental risk of recovery induced by the USA. Figure 6 shows GIRFs of emission pollution in USA, UK, Japan, and China in response to one standard error positive economic shock in United states. The results show that this shock has different implication for the global economy with respect to growth shock in China. USA and China's GIRFs of total $CO_{2}$ emission in response to economic growth in the USA show that $CO_{2}$ pollution in China in response to economic growth in the USA will be significantly different from the pollution emission in the USA. The reason behind this is that any growth in the USA economy, increases the demand for manufactured products produced in China, which are relatively pollutant. Therefore, economic growth in the USA has significant impact on pollution in China. Comparing the result with a positive shock in China shows that pollution emission in response to a positive shock in the USA is much higher than a positive shock in China. According to Kuznets curve, growth in China per capita real GDP is at decreasing stage of the curve, therefore improvement in economic condition after recovery from Covid-19 crisis is not significant for emission pollution. Growth in China's economy includes growth in agricultural products and services which are less pollutant, but growth in the USA economy increases the demand for highly pollutant manufactured products. Our results also show that positive economic shock in the USA has moderate effect on pollution emission in the UK, however its effect on the pollution emission in Japan will be significant. Our results show that a positive economic shock has significant implication for pollution emission in Japan in comparison to other advanced economies. As it can be seen, $CO_{2}$ pollution in Japan will be significantly high, compared to Canada and Australia.

**Figure 7.**
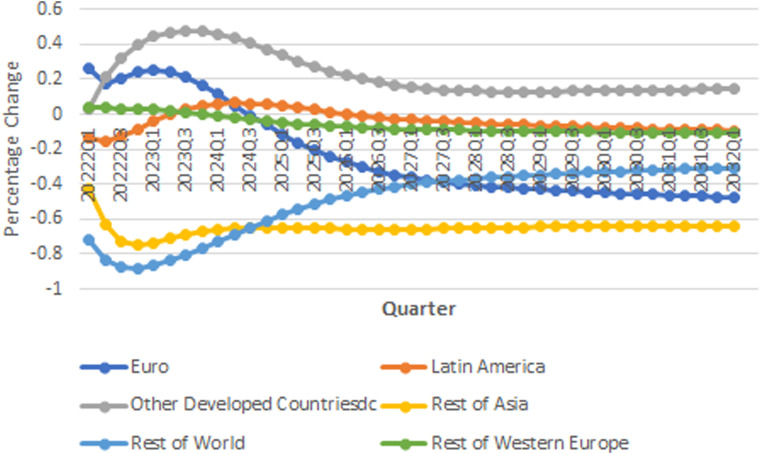
IRFs of $CO_{2}$ emission in response to one standard shock in real GDP in USA.

Figure 7 shows compares total $CO_{2}$ emission pollution in different regions in response to one standard positive economic shock in United States. In Europe, $CO_{2}$ pollution in response to a positive economic growth in the USA will be moderate and decreases over time and it shows that the economic recovery has minimum risk for Europe region. Other Western Europe countries will respond moderately to this shock. In Latin America $CO_{2}$ emission will decrease immediately at the beginning of the recovery of the USA's economy, however $CO_{2}$ pollution response to the economic growth in the USA is heterogeneous within region's countries. Argentina, Mexico, and Chile response positively to a positive shock in the USA's economy. The results also show that in Brazil, $CO_{2}$ pollution will decrease in response to a positive economic growth in the USA economy.

Euro: Austria, Belgium, Finland, France, Germany, Italy, Netherlands, Spain; Rest of Western Europe: Switzerland, Sweden and Norway; Other Developed Countries: Canada, Australia and New Zealand; Latin America: Argentina, Brazil, Chile, Mexico and Peru; Rest of Asia: Indonesia, Korea, Malaysia, Philippine, Singapore and Thailand; Rest of World: India, Turkey, South Africa and Saudi Arabia.

Our results show that pollution emission in Rest of Asia region will decrease in response to economic growth in the USA. This belongs to dependency of these economies on China's economy and the fact that growth in the USA economy replaces China in the word economy and the demand for manufactured products of Asian economies will decrease. Rest of World region will respond negatively to the recovery in the USA economy in short-run, but in long-run pollution emission in these countries will increase in response to a positive shock in the USA economy.

## Discussion and conclusion

Investigations show that during the Covid-19 pandemic, energy consumption and consequently pollution emission reduced at the global level. According to the EKC, any increase in real income can increase emission pollution at the first stage of economic growth, thus economic recovery from the pandemic can increase the level of pollution emission even higher than its pre-pandemic level. Therefore, the world economy is exposed to environmental risk of Covid-19 economic recovery.

Vaccine access remains the principal driver for the global recovery and because of the vaccination coverage, it is expected that the world economy will continue the recovery phase in 2022. However, unequal access to Vaccine, heterogeneous support policies, and future variants of the virus, can have different implications for the recovery at global level. The IMF has forecasted that the world economy grows 4.9 percent in 2022 while growth beyond 2022 is projected to be moderate to 3.3 percent over the mid-term. It is also forecasted that advanced economies experience growth rate higher than pre-pandemic due to sizable policy support in the USA. The IMF has also forecasted a 4.5 and a 6.3 percent growth rate for the USA and China in 2022 respectively. In contrast, emerging economies and developing countries will lose output due to lower rate of vaccination and less policy support compared to advanced economies.

This research applied an E-GVAR model for 33 countries which stands for 90% of world GDP to study the environmental risk of Covid-19 recovery. Results show that positive economic shocks in leading economies such as China and the USA have different implications for the world economy and the environment. A positive economic shock will put the world environment at risk of $CO_{2}$ pollution. The results also show that positive economic shock in China and the USA have different implications for emission pollution at global level. Not only the level of $CO_{2}$ pollution emission in different regions around the world is not the same for these shocks, but also the direction of changes varies in different regions and economies.

Our results show that, China's responses to both shocks are significant and China is exposed to a high environmental risk both because of a positive economic shock in China and the USA, but the level of $CO_{2}$ emission will be much higher in response to a positive economic shock in the USA. On the other hand, The USA response to a positive economic shock in China is lower than the effect of a positive shock in the USA economy. Therefore, USA's economy will be exposed to a higher level of risk in response to its own positive economic shock rather than a positive shock due to the recovery for Covid-19 in China.

Since the IMF has already predicted economic recovery in the USA for the 2022, it is expected that the USA is exposed to environmental risk of the recovery. While Japan response to both shocks is significant, the UK responses to this shock will be moderate. European countries response to both shocks will be moderate and heterogeneous between countries within the European Union and other Western Europe's economies. Other developed countries, including Canada, Australia and New Zealand response to both shock will be similar. Different response of Rest of Asia to positive shocks in China and the USA roots in the structure of industries in this region. Industries in this region interconnected to industries in China. Therefore, this region is exposed to environmental risk of recovery in China's economy. In Latin America, response to both shock will be different. A positive shock in China's economy has positive and moderate impacts on countries in this region. This region responses negatively to a positive shock in the USA's economy in short-run. However, the behavior of countries in the region differs from each other. For example Behavior of Brazil's economy in response to positive shocks in China and the USA is different from other countries within the region. Economies in Rest of World group, response to these shocks is not significantly different. During last decades international cooperation on climate change has become more institutionally diverse which create opportunities to control world emission pollution. Improvement in energy intensity and technical progress potentially can contribute to control emission pollution. Academic researchers can evaluate effectiveness of investment in energy efficiency enhancement and effectiveness of these institutional cooperation to control emission pollution in post Covid-19 period.

## Supplemental Material

sj-xls-1-eae-10.1177_0958305X221108493 - Supplemental material for Environmental risk of Covid-19 recovery Click here for additional data file. Supplemental material, sj-xls-1-eae-10.1177_0958305X221108493 for Environmental risk of Covid-19 recovery by Mortaza Baky Haskuee and Ali Asgary in Energy & Environment
